# Miscellaneous

**Published:** 1887-05

**Authors:** 


### MISCELLANEOUS.

If it were possible to rise above the atmosphere which surrounds the earth, we should see nothing but an intense and sharply-defined ball of fire, while everything else would be wrapped in total darkness. There could be no diffusion of light without an atmosphere or some similar medium to act upon; but if the air about us extended to a height of seven hundred miles, the rays of the sun could not penetrate it, and we would be left in darkness. At the depth of seven hundred feet in the ocean the light ceases altogether, one-half of the light being absorbed in passing through only seven feet of the purest water.

Said She was in Heaven.—A remarkable faith-cure was reported from Banksville, a mining town two miles southwest of Pittsburgh. For several years Maggie Beadling, the sixteen-year-old daughter of a miner, has been bed-ridden. Frequently of late she has laid in a trance or comatose state for days, and when she would return to consciousness she would tell her friends that she had been to heaven. In proof of the story of her transition she told the names and described the appearance of relatives who died before she was born, and of others whom she knew when they were alive. In October and November last she lay in a comatose state for several weeks, during which time she partook only of a small quantity of liquid food administered to her by attendants. When she revived she claimed to have received divine communication to the effect that at two o'clock on the afternoon of February 17th she would be raised from an invalid's bed and entirely restored to health. That afternoon her father's house was filled with friends and curiosity-seekers. Promptly at two o'clock the young girl arose from her chair with her crutches in her hands and, flinging them aside, walked about the room, to the astonishment of those present. The cure seemed complete and she jumped about the room like a child. The news was noised about, and almost the entire populatian of Banksville turned out to see the wonderful case. The parents wept with joy and the greatest excitement prevailed. Miss Beadling was seen by several reporters and she pronounced herself well. It is the belief among neighbors that the young lady was sincere in her claim of affliction, and her recovery is regarded as a miracle.—*World, Feb.* 18, 1887.

Scientific.—At a meeting of the Physicological Society of Berlin, in February last, Dr. Mullenhoff referred to a treatise by Kepler, that appears to have been forgotten, on the structure of the cells of bees. A fact was communicated to the meeting which is very interesting and new, though possibly already known to some readers. It is that, when the bee has filled the cell either with pure honey, or a mixture of pollen-dough and honey, and has completed the lid, a drop of formic acid obtained from the poison bag connected with the sting is added to the honey by perforating the lid with the sting. Numerous experiments show that this formic acid preserves honey and every other sugar solution from fermentation. If this be well established, it will show that the sting and the poison apparatus of the bee has a further purpose than that of a defensive or offensive weapon. Another interesting fact suggests itself in connection with this. So far as is known, most of the insects that have stinging apparatus similar to that of the bee are collectors and storers of honey.—*Science Gossip.*

A TRIBUTE TO CALIFORNIA.—A Chicago correspondent of the Worcester (Mass.) *Herald*, in his description of the exhibition of citrus fruits from Southern California in the city by the lake, says: "California, with its 160,000 square miles of territory, its eight hundred miles of sea coast, its grand Yosemite valley; its stupendous waterfalls; its giant trees, its towering mountains, presents within the limits of a single State all the climates known to the union; all the differences of surface from snow-clad peaks to valleys which lie hundreds of feet below the sea level; all fruits between the equator and the pole; all the minerals known to geology. She invites the world to her table and all may be filled. Her ships go forth to the ends of the earth, laden with gold and with grain; with wool and wine; with oranges and oil; with cattle and coin. She has added more than a thousand million dollars to the wealth of the world, in gold alone, and the end is yet afar off. But it is not the beauty of scenery, nor the gold from her mines, which will make her future fame; it will be the grain from her wheat fields, the fruit from her citrus groves, the wine from her vineyards, the wool from her flocks, the cattle from her hills, the spice-laden breezes which fill her sanitariums with health-seekers, the rose-clad homes which shelter her workers; these will be her glory and make her enduring fame.—*Western Trail.*

### THEN AND NOW.

### THEN.

A sombre room, the curtains drawn,
No outward look upon the lawn,
No freshening air nor cheerful ways,
A mother's ever anxious gaze.

A step upon the outer stair,
A presence and a business air.

O, Doctor, I'm so glad you're here.
It seemed so long—so long to wait
Alone with grief and death so near—
So very near, and you so late.

Is there no change!

My darling child!
It is as if the angels smiled
Upon our hope, for now she sleeps.
While yet the crimson current leaps

Too wildly to her fevered brow—
She begged for water even now
With such a burning, parching thirst,
Not all my strength could well resist.

Water! not so, 'twere worse than mad.
Fetch here a bowl, she must be bled.

Bled, Doctor! *she*, so faint and low
And fever worn—oh, say not so.

*She must be bled.* Such is the rule
And practice of our famous school.

'Twere done, and ere another morn
A childless mother wept, forlorn,
And when, with reverend hands, they laid
The little sufferer neath the sod,
With sorrowing hearts they meekly said
"*Amen. It was the will of God.*"

NOW.

The scene is new. A sunny room
With light and air and flowers in bloom,
Where on her couch an infant lies
With benedictions in her eyes,
As to her lips the cup is held
Of nature's pearly fountain filled.

No health-forbidding torture now,
No piercing lance and life-blood flow.
For wiser heads have learned the art
Of helping nature with her part,
And *she* provides for every harm
If we but heed, a healing balm.
And one to read her book aright
Hath little need of other light.
And yet the schoolmen and the quack
Alike pursue a beaten track,
And if the patient live, they're sure
To take the credit of a cure.
But if he die of drug or prod,
They charge the debit side to *God.*

